# Different profiles of lipoprotein particles associate various degrees of cardiac involvement in adolescents with morbid obesity

**DOI:** 10.3389/fped.2022.887771

**Published:** 2022-11-22

**Authors:** José M. Siurana, Anna Sabaté-Rotés, Núria Amigó, Neus Martínez-Micaelo, Larry Arciniegas, Lucia Riaza, Eduard Mogas, Ferran Rosés-Noguer, Paula S. Ventura, Diego Yeste

**Affiliations:** ^1^ Department of Pediatric Cardiology, Hospital HM Nens, HM Hospitales, Barcelona, Spain; ^2^ Autonomous University of Barcelona, Barcelona, Spain; ^3^ Department of Pediatric Cardiology, Vall d’Hebron University Hospital, Barcelona, Spain; ^4^ Biosfer Teslab, Reus, Spain; ^5^ Department of Basic Medical Sciences, Universitat Rovira I Virgili, Institut D'Investigació Sanitària Pere Virgili (IISPV), Tarragona, Spain; ^6^ Centro de Investigación Biomédica en Red de Diabetes y Enfermedades Metabólicas Asociadas (CIBERDEM), Instituto de Salud Carlos III (ISCIII), Madrid, Spain; ^7^ Department of Pediatric Endocrinology, Vall d'Hebron University Hospital, Barcelona, Spain; ^8^ Department of Pediatric Radiology, Vall d'Hebron University Hospital, Barcelona, Spain; ^9^ Department of Pediatric Endocrinology, Hospital HM Nens, HM Hospitales, Barcelona, Spain; ^10^ Fundació Institut d’Investigació en Ciències de la Salut Germans Trias i Pujol (IGTP), Badalona, Spain; ^11^ Centro de Investigación Biomédica en Red de Enfermedades Raras (CIBERER), Instituto de Salud Carlos III (ISCIII), Madrid, Spain

**Keywords:** morbid obesity, adolescents, lipoprotein subclasses, atherosclerotic phenotype, metabolic syndrome, cardiac changes, systolic dysfunction, small LDL particles

## Abstract

**Introduction:**

Dyslipidemia secondary to obesity is a risk factor related to cardiovascular disease events, however a pathological conventional lipid profile (CLP) is infrequently found in obese children. The objective is to evaluate the advanced lipoprotein testing (ALT) and its relationship with cardiac changes, metabolic syndrome (MS) and inflammatory markers in a population of morbidly obese adolescents with normal CLP and without type 2 diabetes mellitus, the most common scenario in obese adolescents.

**Methods:**

Prospective case-control research of 42 morbidly obese adolescents and 25 normal-weight adolescents, whose left ventricle (LV) morphology and function had been assessed. The ALT was obtained by proton nuclear magnetic resonance spectroscopy, and the results were compared according to the degree of cardiac involvement – normal heart, mild LV changes, and severe LV changes (specifically LV remodeling and systolic dysfunction) – and related to inflammation markers [highly-sensitive C-reactive protein and glycoprotein A (GlycA)] and insulin-resistance [homeostatic model assessment for insulin-resistance (HOMA-IR)]. A second analysis was performed to compare our results with the predominant ALT when only body mass index and metabolic syndrome criteria were considered.

**Results:**

The three cardiac involvement groups showed significant increases in HOMA-IR, inflammatory markers and ALT ratio LDL-P/HDL-P (40.0 vs. 43.9 vs. 47.1, *p* 0.012). When only cardiac change groups were considered, differences in small LDL-P (565.0 vs. 625.1 nmol/L, *p* 0.070), VLDL size and GlycA demonstrated better utility than just traditional risk factors to predict which subjects could present severe LV changes [AUC: 0.79 (95% CI: 0.54–1)]. In the second analysis, an atherosclerotic ALT was detected in morbidly obese subjects, characterized by a significant increase in large VLDL-P, small LDL-P, ratio LDL-P/HDL-P and ratio HDL-TG/HDL-C. Subjects with criteria for MS presented overall worse ALT (specially in triglyceride-enriched particles) and remnant cholesterol values.

**Conclusions:**

ALT parameters and GlycA appear to be more reliable indicators of cardiac change severity than traditional CV risk factors. Particularly, the overage of LDL-P compared to HDL-P and the increase in small LDL-P with cholesterol-depleted LDL particles appear to be the key ALT's parameters involved in LV changes. Morbidly obese adolescents show an atherosclerotic ALT and those with MS present worse ALT values.

## Introduction

Cardiovascular diseases (CVD) are the main cause of death in the world (1) and, if the trend initiated in 2,000 continues, CVD will also become the main cause of death among contemporary children. Obesity is a multifactorial disease that includes several preceding disorders of CVD, such as high blood pressure (BP), insulin resistance, diabetes and dyslipidemia. However, the majority of obese children present a pre-pathological condition, therefore identifying which of them are at increased risk of developing CVD is a challenge for pediatricians.

In the conventional lipid profile (CLP), the increase in cholesterol in low-density lipoproteins (LDL-C) has been demonstrated as one of the most important factors associated with CVD risk (2), but the role of high-density lipoprotein cholesterol (HDL-C) or triglycerides (TG) remains unclear (3). Additionally, the remnant cholesterol – a derivative from the CLP that accounts for the cholesterol enclosed in the very low-density lipoproteins (VLDL), intermediate-density lipoproteins (IDL) and chylomicrons – has been also associated with increased prevalence of ischemic heart disease (4) and myocardial infarction (5). On the other hand, the advanced lipoprotein testing (ALT), assessed by proton nuclear magnetic resonance spectroscopy (1H-NMR) of plasma, provides much information about lipoprotein characteristics as it quantifies the concentration, particle size and composition (cholesterol or TG) of each lipoprotein subclass. Previous studies have shown how in situations of apparent normality, specific lipoprotein alterations are highly associated with future CVD events (6–9). Thus, the concept of atherogenic dyslipidemia – applied to subjects with hypo HDL-C, hypertriglyceridemia and normal LDL-C, but increases in some ALT components – has been associated with an elevated risk of CVD events (10–12).

Various studies have evaluated the ALT in obese adults and adolescents and established its utility in predicting arterial wall changes, coronary heart disease or CVD events (13–15), however to our knowledge, none have analyzed which ALT components are closely associated with cardiac changes in obese adolescents. Our group showed in a previous publication that morbidly obese adolescents presented left ventricular remodeling and systo-diastolic dysfunctions, closely related to body mass index (BMI) (16). The objective of the current study is to evaluate the ALT and its relationship with cardiac changes, metabolic syndrome (MS) and inflammatory markers in the same cohort of morbidly obese adolescents with normal CLP and without type 2 diabetes mellitus (T2-DM), the most common scenario in obese adolescents.

## Methods

### Study population

Prospective observational case-control research of 67 adolescents of both genders between 10 and 17 years old. Subjects with morbid obesity were recruited from endocrinology clinics and control participants were recruited from healthy volunteers in the cardiology and sports medicine clinics.

Subjects with current infectious or recent acute inflammatory processes, history of prematurity or birth weight <2,000 g, and smokers or those whose pathologies could affect the cardiovascular system – such as congenital heart disease, chronic kidney disease, transplant, rheumatic diseases, and HIV infection – were excluded. Similarly, obese subjects with T2-DM and control subjects who practiced >7 weekly sport hours, a threshold explained in our previous study (16), were also excluded in this analysis.

Subjects were classified depending on the degree of cardiac involvement and three groups were established: no cardiac changes (*n* 25), formed by control and obese adolescents without LV changes; mild cardiac changes (*n* 17), consisting of 15 obese adolescents with LV remodeling and 2 control adolescents with border systolic dysfunction; and severe cardiac changes (*n* 25), constituted by morbidly obese adolescents with LV remodeling and systolic dysfunction, based on the threshold below defined. For the second analysis, subjects were reclassified according to their BMI, calculated from tables of the Barcelona Longitudinal Growth Study 1995–2017 (17), in two groups: subjects with morbid obesity (SDS-BMI ≥4; *n* 42), and with normal weight (BMI 5th–85th percentile, *n* 25). Finally, the last classification of subjects was performed on the base of MS criteria from Cook et al. (18), identifying two groups: subjects without MS (*n* 53), and with MS (*n* 14).

Written informed consent for participation was obtained and the Institutional Review Board at Vall d'Hebron Hospital approved the protocol (PR-AMI-273/2018). All subjects provided assent and an informed consent was signed by their parents/legal guardians.

### Clinical and laboratory assessment

Demographic data of age, gender and clinical status were obtained from patient anamnesis. Blood pressure was obtained using a Welch Allyn Spot Vital Signs Monitor (4200B, Hillrom, Batesville, Indiana) after subjects had rested for 5 min, in a supine position and with an appropriately sized cuff giving measured mid-arm circumference, according to the criteria of the European Society of Hypertension (19), which also defines the high BP values by age and gender.

EDTA blood samples were obtained at the time of enrollment at the participating hospitals in the morning after at least 8 h of fasting. Samples for lipoprotein particle analysis were aliquoted, stored in liquid nitrogen (−80°C) and shipped on dry ice to Biosfer Teslab, where the ALT was measured by 1H-NMR spectroscopy, based on the LipoScale test® (20). For each main lipoprotein class (VLDL, LDL and HDL) we obtained large, medium and small subclass particle concentrations (VLDL-P, LDL-P, HDL-P), mean particle size and composition (ratio VLDL-TG/VLDL-C, ratio LDL-TG/LDL-C, ratio HDL-TG/HDL-C). Additionally, tests performed on blood samples from all subjects were: fasting glycemia and insulin, glycated hemoglobin and the CLP (total cholesterol, HDL-C, LDL-C and TG). To evaluate insulin resistance, the homeostasis model assessment of insulin resistance (HOMA-IR) was calculated using the equation: fasting insulin (µU/ml) × fasting glucose (mmol/L)/22.5. Inflammation markers evaluated were highly-sensitive C-reactive protein (hs-CPR) and an NMR derived glycoprotein biomarker, termed glycoprotein A (GlycA), arising from the concentration of the acetyl groups of N-acetylglucosamine and N-acetylgalactosamine bond to plasmatic proteins. Glyc A is able to detect low-grade chronic inflammation in obesity and insulin resistance's disorders (21–23), and in atherosclerosis progression (24).

### Echocardiographic image acquisition and analysis

Patients were examined using a Vivid S60 commercial ultrasound scanner (GE Vingmed Ultrasound AS, Horten, Norway) with a phased-array transducer (GE 3-MHz; GE Vingmed Ultrasound AS). Images were obtained at rest in the supine or left lateral decubitus position in the standard tomographic views of the LV (parasternal long and short axis and apical 4-chamber, 2-chamber, and long-axis views). All echocardiographic images were obtained prospectively by an experienced pediatric cardiologist, according to the criteria of the American Society of Echocardiography (ASE) (25).

To evaluate the LV geometry, relative wall thickness (RWT) and LV mass were calculated using LV linear dimensions and following the recommendation of ASE (25). The LV mass was determined by the adjusted Devereux's equation and the resultant value was indexed to height to the power of 2.7 (LVMI, g/m^2.7^). LV geometry was categorized as normal or pathological (concentric remodeling, eccentric hypertrophy and concentric hypertrophy) considering the following cutoff values (95th percentile in the pediatric population): LVMI >45 g/m^2.7^ in males and >40 g/m^2.7^ in females (26), and RWT >0.41 (27).

The LV function was determined by two-dimensional speckle tracking echocardiography, a well-validated and precise method to quantify ventricular function with lower variability than LV ejection fraction in pediatric patients (28). Strain and strain rate (SR) were calculated according to the criteria of the ASE (25) and the European Association of Cardiovascular Imaging (28). Two-dimensional video loops were obtained for each patient in apical four, three, and two-chamber views, acquiring images at a frame rate of >65 frames/s. Offline image processing was performed using EchoPAC (version 11.2, GE Vingmed Ultrasound, Horten, Norway). After manually tracing along the endomyocardial border, the software automatically generated the epicardial border and the six segments, which were accepted after a visual inspection. To determine SR and midline strain, at least 17 out of 18 segments had to be included. Measured parameters were LV end-systolic global longitudinal strain (GLS, %) and LV early diastolic global longitudinal SR (early GLSR, 1/sec). Resultant values were calculated by adding the strain of all accepted segments and dividing the value by the total number of segments. The GLS cutoff value which defined systolic dysfunction in this study was −16.7%, which corresponds to the lowest GLS value reported in a meta-analysis of LV strain measures by echocardiography in children (29).

### Statistical analysis

Data from the study were analyzed using descriptive statistics. Quantitative results were expressed as median and 25–75 interquartile range, while qualitative or dichotomous variables were expressed as percentages. Chi-square test (*χ*^2^) and Fisher's exact test were used, according to the size and characteristic of qualitative variables, to compare proportions and to study relationships between them. The comparison between quantitative variables was made by nonparametric Mann–Whitney *U* test or nonparametric Kruskal–Wallis test followed by Dunn's *post hoc* test for multiple comparisons. To estimate correlations between parameters, the Pearson and Spearman's Rho correlation coefficients were calculated as appropriate for the type of the data. Data was analyzed by using IBM SPSS Statistics for Windows, version 23.0 (IBM Corp., Armonk, N.Y.) and values of *p* < 0.05 were considered significant.

A three-step multivariate analysis was applied to identify important variables and patterns that allowed distinguishing between the three cardiac involvement groups. In the first step, we applied three statistical approaches to identify the variables that make the largest contributions to the discrimination between groups. These approaches include the Wilcoxon rank-sum test, the Random Forest, and the Partial Least Squares discriminant analysis (PLS-DA). Those variables that resulted significant with a *p*-value <0.05 were selected as the candidate for the Wilcoxon rank-sum test, and the 5–10 most important variables were determined by the variable importance score or the variable importance in projection (VIP) score using the Random Forest or the PLSA-DA, respectively. To avoid overfitting, 10-fold cross-validation was performed. In the second step, by using a Venn diagram we selected the most prominent variables and those that will be included in the model by determining those that overlap by the statistical approaches. In the third step, we used the Principal Component Analysis (PCA) as an unsupervised method to visualize the capacity of the selected variables to drive group separation. Ellipses in PCA represent 90% confidence intervals around the centroid of each data cluster. Finally, we built a linear fitting model, and by computing the area under the curve (AUC) and the 95% confidence interval of a receiver operating characteristics (ROC) curve we evaluated and quantified how accurately the selected variables were able to discriminate between groups. Patients were randomly assigned to training (60%) and test (40%) sets. We performed 10-fold cross-validation with 100 replicates on the training data during the model construction process and tested the model on the hold-out data. Analysis was performed using the R statistical software version 4.1.1 (Chapman & Hall/CRC Computational Biology Series). Additionally, a logistic regression was performed to model the probability of dichotomous events (mild or severe cardiac changes, and no changes or severe cardiac changes) with the selected variables in the previous statistical analysis.

## Results

**Figure 1:**
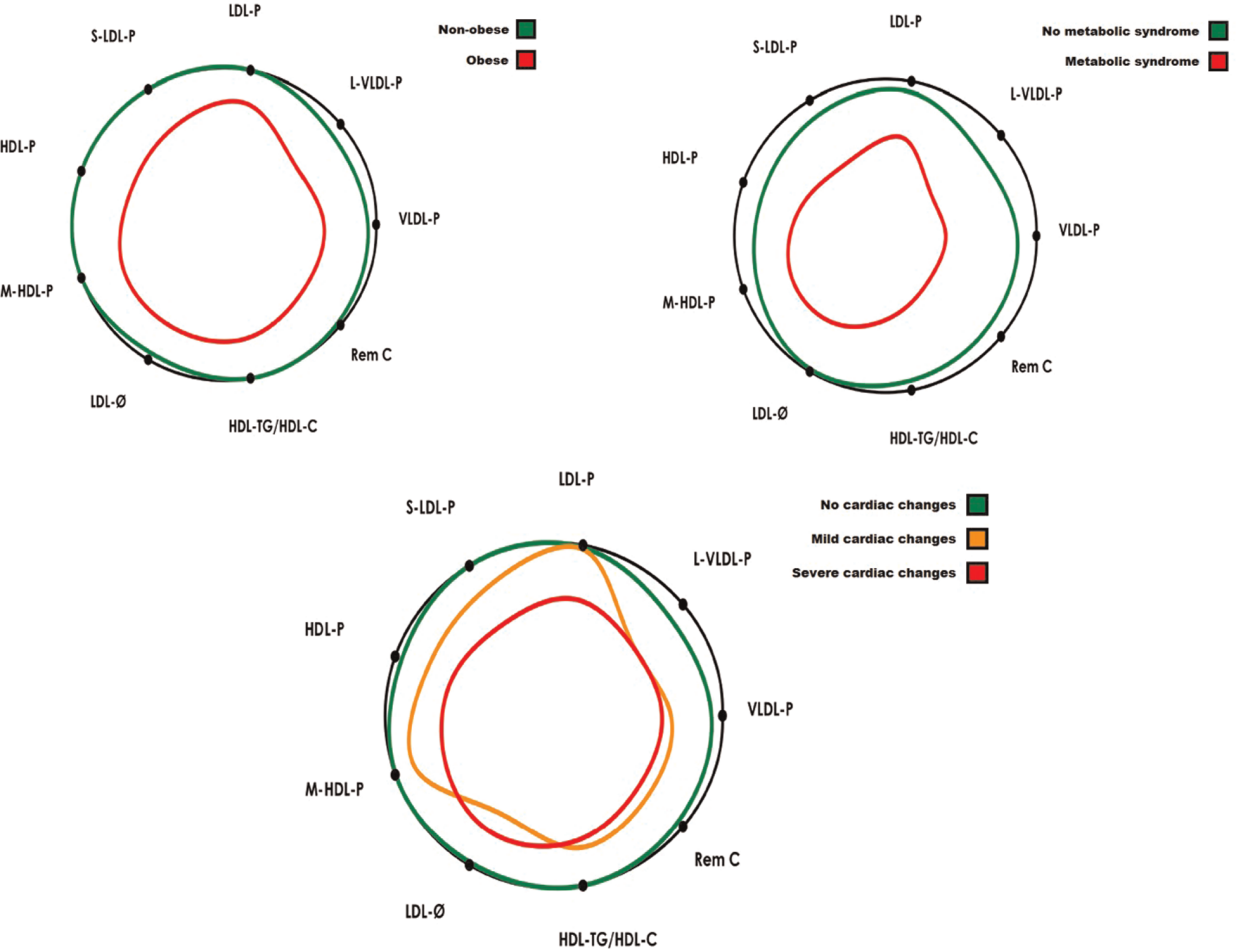
The lipid contour is a graphical model to facilitate the lipoprotein profile interpretation and its association with cardiovascular risk. The colored silhouettes represent the patient groups’ values with respect to the values of an apparently healthy population (black circle) (30). The study group's contour delimits a smaller central area when the variables have values associated with an increased risk of developing CVD (i.e., values higher than the reference population’s mean for L-VLDL-P, VLDL-P, Rem-C, S-LDL-P, LDL-P, HDL-TG/HDL-C variables; or lower than reference population’s mean for LDL-Ø, HDL-P and M-HDL-P variables). Colored silhouettes are represented in percentage of increase or reduction regarding data in Tables 1–3. **Bottom of the figure:** VLDL-P, total VLDL particles; L-VLDL-P, large VLDL particles; LDL-P, total LDL particles; S-LDL-P, small LDL particles; HDL-P, total HDL particles; M-HDL-P, medium HDL particles; LDL-Ø, LDL particles diameter; HDL-TG/HDL-C, triglyceride enriched HDL; Rem C, remnant cholesterol.

A total of 42 adolescents with obesity and 25 adolescents without obesity, age-matched, were included in our study. No subject was excluded, although HbA1c was not registered in ten subjects of the obese group and hs-CPR values were missed in one control and three obese subjects. Main clinical, laboratory and echocardiographic data from the three cardiac groups are summarized in Table 1. No significant differences were noted in age, but most obese subjects were classified in the mild or severe cardiac change groups. However, the significant increase observed in BP, HOMA-IR and inflammation markers among the three groups suggests a relationship between the severity of cardiac changes and the worsening in CV risk factors, except in the conventional lipid profile, where no differences were found among both groups with cardiac changes. In contrast, significant differences were detected in the ALT (Table 1), and a pathological phenotype related to cardiac changes was defined (Figure 1): increases in concentrations of total VLDL-P, large VLDL-P, total LDL-P, small LDL-P, ratio LDL-P/HDL-P, and ratio HDL-TG/HDL-C, and decreases in concentrations of total HDL-P and medium HDL-P, and in the LDL size.

**Table 1:** Clinical and laboratory characteristics of subjects depending on cardiac involvement degree groups.

**Variable**	**No cardiac disorder**	**Mild cardiac changes**	**Severe cardiac changes**	*P* value			**P value** **No vs Mild vs Severe**
	(*N* = 25)	(*N* = 17)	(*N* = 25)	**No vs Mild**	**No vs Severe**	**Mild vs Severe**	
**Age (years)**	14 [13-15]	14 [13-16]	14 [13-15]	0.179	0.743	0.239	0.352
**BMI (kg/m^2^)**	19.5 [17.9-22.7]	35.2 [32.7-38.9]	37.8 [34.9-40.2]	<0.001	<0.001	0.238	<0.001
**BMI SD**	-0.1 [-0.9-1.1]	7.0 [5.9-8.4]	7.8 [6.4-9.0]	<0.001	<0.001	0.303	<0.001
**SBP (mmHg)**	113 [101-119]	120 [114-128]	126 [119-141]	0.011	<0.001	0.178	<0.001
**DBP (mmHg)**	64 [57-69]	66 [59-76]	76 [67-81]	0.307	0.001	0.040	0.002
**BP ≥p90 (%)**	12	35	68	0.124	<0.001	0.059	<0.001
**LABORATORY PARAMETERS**							
Fasting glucose (mg/dL)	79 [71-84]	82 [72-90]	83 [78-88]	0.279	0.022	0.662	0.094
HbA1c (%) (N=57)	5.2 [5.1-5.5]	5.3 [5.2-5.5]	5.4 [5.2-5.6]	0.291	0.172	0.650	0.315
HOMA-IR	1.4 [1.0-2.3]	3.6 [1.8-5.0]	6.6 [3.8-8.2]	0.019	<0.001	0.013	<0.001
**Classical lipid profile**							
Total Cholesterol (mg/dL)	173 [164-188]	181 [143-201]	179 [169-198]	0.749	0.282	0.729	0.610
LDL-C (mg/dL)	103 [91-113]	98 [77-120]	106 [96-121]	0.635	0.327	0.254	0.433
HDL-C (mg/dL)	52 [45-60]	44 [41-55]	44 [41-48]	0.048	0.001	0.283	0.003
Triglycerides (mg/dL)	71 [60-92]	95 [75-118]	87 [74-140]	0.026	0.010	0.933	0.018
Remnant cholesterol (mg/dL)	18.9 [13.5-22.5]	24.2 [17.5-30.3]	22.0 [18.1-36.9]	0.037	0.019	0.977	0.032
HDL-C ≤40 mg/dL (%)	0	18	14	0.059	0.235	0.672	0.115
Triglycerides ≥150 mg/dL (%)	0	12	16	0.158	0.110	1.000	0.126
**Inflammatory markers**							
Highly sensitive C-reactive protein (mg/dL) (N=63)	0.02 [0.01-0.09]	0.12 [0.06-0.24]	0.24 [0.14-0.52]	0.012	<0.001	0.041	<0.001
Glycoprotein A (µmol/L)	618 [583-747]	707 [659-830]	845 [731-982]	0.030	<0.001	0.034	<0.001
**Lipoprotein Particles**							
**VLDL-P (nmol/L)**							
Total	30.1 [25.5-40.7]	45.0 [29.3-55.1]	37.4 [31.6-69.5]	0.041	0.007	0.691	0.016
Large	0.8 [0.7-1.2]	1.3 [0.9-1.6]	1.1 [0.9-1.6]	0.024	0.007	0.866	0.013
Medium	3.3 [2.6-5.0]	4.7 [3.6-8.0]	5.0 [2.7-6.2]	0.022	0.114	0.635	0.070
Small	26.1 [22.7-33.6]	39.3 [25.0-47.3]	30.1 [27.6-59.9]	0.046	0.005	0.596	0.014
**LDL-P (nmol/L)**							
Total	1023.6 [913.5-1131.3]	1017.8 [818.1-1182.7]	1123.0 [1015.8-1245.2]	0.868	0.034	0.115	0.086
Large	165.0 [151.6-174.9]	163.8 [129.8-188.7]	172.3 [147.3-183.0]	0.582	0.720	0.497	0.752
Medium	300.4 [248.3-356.3]	299.0 [146.3-378.2]	335.7 [257.6-381.7]	0.635	0.290	0.244	0.402
Small	560.0 [517.1-599.2]	565.0 [522.8-639.0]	625.1 [551.7-685.3]	0.390	0.003	0.070	0.011
**HDL-P (µmol/L)**							
Total	26.5 [23.7-29.3]	24.3 [20.6-29.0]	24.2 [22.1-25.6]	0.244	0.015	0.547	0.064
Large	0.22 [0.2-0.3]	0.26 [0.2-0.3]	0.25 [0.2-0.3]	0.001	0.035	0.155	0.003
Medium	9.3 [8.6-10.4]	9.0 [8.1-10.3]	8.9 [7.9-9.3]	0.363	0.034	0.391	0.112
Small	16.8 [14.5-18.9]	15.2 [12.0-19.2]	14.8 [13.2-16.4]	0.405	0.029	0.599	0.129
**Size (nm)**							
VLDL size	42.3 [42.0-42.4]	42.4 [42.3-42.4]	42.1 [42.0-42.3]	0.337	0.218	0.037	0.106
LDL size	21.1 [20.9-21.2]	21.0 [20.7-21.2]	21.0 [20.9-21.1]	0.060	0.049	0.635	0.072
HDL size	8.3 [8.2-8.3]	8.3 [8.2-8.4]	8.3 [8.2-8.3]	0.434	0.233	0.990	0.488
**Composition**							
Ratio VLDL-TG/VLDL-C	3.70 [3.4-4.0]	3.62 [3.5-4.1]	3.9 [3.5-4.2]	0.929	0.367	0.513	0.643
Ratio IDL-TG/IDL-C	1.20 [1.1-1.4]	1.20 [1.1-1.4]	1.10 [1.1-1.2]	0.868	0.148	0.163	0.245
Ratio LDL-TG/LDL-C	0.11 [0.1-0.1]	0.12 [0.1-0.1]	0.14 [0.1-0.2]	0.660	0.006	0.044	0.017
Ratio HDL-TG/HDL-C	0.23 [0.2-0.3]	0.29 [0.2-0.4]	0.30 [0.2-0.4]	0.011	0.005	0.992	0.007
**Ratio LDL-P/HDL-P**	40.0 [31.7-47.6]	43.9 [34.4-45.9]	47.1 [40.7-53.1]	0.646	0.005	0.037	0.012
**Non-HDL-P (nmol/L)**	1034.7 [934.9-1148.1]	1122.0 [837.8-1228.5]	1153.1 [1042.3-1282.1]	0.929	0.013	0.141	0.053

Values expressed in median and 25-75% IQR; P value determined using Kruskal-Wallis test followed by Dunn’s post hoc test for multiple comparisons; Dichotomous variables (Fisher exact test); BMI, Body Mass Index; SD, Standard deviation; SBP, Systolic blood pressure; DBP, Diastolic blood pressure; HOMA-IR, Homeostatic model assessment insulin resistance.

Data of LV geometry and speckle tracking determinations from the echocardiographic evaluation are summarized in Supplementary Table S1.

Table 2 shows the clinical characteristics, laboratory parameters and ALT of subjects classified by obesity index. The obese subjects exhibited, in addition to the pathological ALT phenotype observed in subjects with cardiac changes, a mild, though significant, difference in the concentration of HDL-C (52 vs. 44 mg/dl, *p* < 0.001), triglycerides (77 vs. 91 mg/dl, *p* < 0.001) and remnant cholesterol (19.1 vs. 23.9 mg/dl, *p* 0.010) (Figure 1). Finally, data of subjects categorized by MS criteria are summarized in Table 3. Obese adolescents who accomplished MS criteria showed overall worse BP, insulin resistance and hs-CRP values than any of the previous groups. Their CLP was characterized by marked differences in triglycerides (78 vs 124 mg/dL, *p* < 0.001) and remnant cholesterol (19.6 vs 30.3 mg/dL, *p* < 0.001), while the ALT was characterized by significant increases in: total VLDL-P, large VLDL-P, ratio LDL-P/HDL-P and ratio HDL-TG/HDL-C (Figure 1). The resultant ALT did not differ when only obese subjects were categorized by MS criteria, remarking the central role of hypertriglyceridemia in subjects with MS (Supplemental Table S2).

**Table 2:** Clinical and laboratory characteristics of subjects depending on body mass index groups.

Variable	Non-obese (*N* = 25)	Obese (*N* = 42)	*p* value
**Age (years)**	14 [13-15]	14 [13-15]	0.726
**Male**	52% (N=13)	33% (N=14)	0.198
**BMI (kg/m^2^)**	19.4 [17.9-22.1]	36.8 [34.3-39.8]	<0.001
**BMI SD**	-0.2 [-0.9-0.7]	7.5 [6.4-8.5]	<0.001
**Waist circumference (cm)**	69 [65-74]	111 [102-120]	<0.001
**SBP (mmHg)**	108 [101-118]	124 [118-134]	<0.001
**DBP (mmHg)**	60 [60-68]	74 [65-78]	<0.001
**LABORATORY PARAMETERS**			
Fasting glucose (mg/dL)	76 [71-84]	83 [75-89]	0.012
HbA1c (%) (*N* = 57)	5.2 [5.1-5.5]	5.3 [5.2-5.5]	0.218
HOMA-IR	1.2 [1.0-1.7]	5 [3.6-7.9]	<0.001
**Classical lipid profile**			
Total Cholesterol (mg/dL)	180 [164-191]	178 [156-197]	0.613
LDL-C (mg/dL)	102 [90-115]	105 [91-118]	0.604
HDL-C (mg/dL)	52 [48-61]	44 [41-49]	<0.001
Triglycerides (mg/dL)	77 [60-90]	91 [72-130]	0.006
Remnant cholesterol (mg/dL)	19.1 [14.1-22.5]	23.9 [18.0-36.3]	0.010
**Inflammatory markers**			
Highly-sensitive C-reactive protein (mg/dL)	0.02 [0.01-0.05]	0.22 [0.13-0.45]	<0.001
Glycoprotein A (µmol/L)	620 [583-687]	802 [705-938]	<0.001
**LIPOPROTEIN PARTICLES**			
**VLDL-P (nmol/L)**			
Total	30.0 [25.5-40.7]	42.8 [31.5-63.7]	0.002
Large	0.8 [0.7-1.2]	1.3 [0.9-1.6]	0.001
Medium	3.7 [2.8-5.4]	4.6 [3.0-6.5]	0.072
Small	26.1 [22.4-33.6]	37.3 [27.3-55.6]	0.002
**LDL-P (nmol/L)**			
Total	1023.6 [904.7-1136.9]	1096.5 [977.4-1196.7]	0.133
Large	165.1 [150.6-183.3]	164.4 [145.2-182.5]	0.726
Medium	300.4 [225.2-356.3]	332.1 [249.9-384.6]	0.371
Small	560.0 [517.1-607.0]	616.2 [527.3-676.3]	0.019
**HDL-P (µmol/L)**			
Total	27.0 [24.2-30.1]	24.2 [21.7-26.4]	0.001
Large	0.2 [0.2-0.3]	0.2 [0.2-0.3]	0.010
Medium	9.6 [8.7-10.9]	8.9 [7.9-9.4]	0.018
Small	17.3 [14.8-19.2]	17.8 [12.8-17.0]	0.005
**Size (nm)**			
VLDL size	42.3 [42.2-42.4]	42.2 [42.0-42.4]	0.371
LDL size	21.1 [21.0-21.2]	21.0 [20.9-21.1]	0.058
HDL size	8.3 [8.2-8.3]	8.3 [8.2-8.3]	0.123
**Composition**			
Ratio VLDL-TG/VLDL-C	3.7 [3.4-3.9]	3.9 [3.5-4.2]	0.249
Ratio IDL-TG/IDL-C	1.2 [1.1-1.4]	1.1 [1.1-1.3]	0.042
Ratio LDL-TG/LDL-C	0.1 [0.1-0.1]	0.1 [0.1-0.2]	0.023
Ratio HDL-TG/HDL-C	0.2 [0.2-0.3]	0.3 [0.2-0.4]	0.002
**Ratio LDL-P/HDL-P**	37.1 [31.7-45.2]	46.2 [38.9-51.8]	0.001
**Non-HDL-P (nmol/L)**	1034.7 [915.5-1163.1]	1127.8 [1002.7-1245.4]	0.068

Values expressed in median and 25-75% IQR; P value calculated by non-parametric Mann-Whitney U test; Dichotomous variables (Fisher exact test); BMI, Body Mass Index; SD, Standard deviation; SBP, Systolic blood pressure; DBP, Diastolic blood pressure; HOMA-IR, Homeostatic model assessment insulin resistance.

**Table 3:** Clinical and laboratory characteristics of subjects depending on metabolic syndrome (MS) diagnosis.

Variable	No MS (<3 factors) (*N* = 53)	MS (≥3 factors) (*N* = 14)	*p* value
**Age (years)**	14 [13-15]	14 [13-15]	0.825	
**BMI (kg/m^2^)**	32.1 [19.4-37.0]	38.1 [35.9-43.0]	<0.001	
**BMI SD**	5.7 [-0.1-7.5]	7.9 [6.5-10.3]	0.001	
**SBP (mmHg)**	118 [106-122]	131 [126-144]	<0.001	
**DBP (mmHg)**	67 [59-74]	77 [69-80]	0.002	
**LABORATORY PARAMETERS**				
Fasting glucose (mg/dL)	79 [72-86]	84 [82-89]	0.024	
HbA1c (%) (*N* = 57)	5.3 [5.1-5.5]	5.4 [5.1-5.6]	0.515	
HOMA-IR	2.3 [1.3-4.7]	7.4 [4.2-8.9]	<0.001	
**Classical lipid profile**				
Total Cholesterol (mg/dL)	176 [164-193]	188 [164-207]	0.185	
LDL-C (mg/dL)	103 [90-117]	105 [95-121]	0.633	
HDL-C (mg/dL)	49 [43-56]	42 [36-47]	0.003	
Triglycerides (mg/dL)	78 [64-99]	124 [103-163]	<0.001	
Remnant cholesterol (mg/dL)	19.6 [14.6-24.1]	30.3 [25.2-44.4]	<0.001	
**Inflammatory markers**	** **	** **	** **
Highly sensitive C-reactive protein (mg/dL)	0.09 [0.02-0.21]	0.37 [0.15-0.50]	0.001
Glycoprotein A (µmol/L)	701 [616-787]	868 [777-999]	0.001
**LIPOPROTEIN PARTICLES**			** **
**VLDL-P (nmol/L)**			** **
Total	31.7 [26.9-45.0]	59.6 [47.6-82.5]	<0.001
Large	0.9 [0.7-1.3]	1.6 [1.3-1.8]	<0.001
Medium	3.7 [2.6-5.4]	6.1 [4.5-9.4]	<0.001
Small	28.3 [23.3-38.6]	52.2 [40.1-71.4]	<0.001
**LDL-P (nmol/L)**			
Total	1033.2 [913.5-1175.2]	1092.5 [998.0-1256.9]	0.235
Large	165.1 [146.0-183.3]	161.1 [147.8-180.5]	0.841
Medium	311.5 [232.6-368.0]	347.7 [256.5-377.1]	0.600
Small	575.8 [529.4-627.0]	644.2 [518.6-714.6]	0.112
**HDL-P (µmol/L)**	** **	** **	** **
Total	25.5 [23.0-28.7]	23.5 [20.3-26.0]	0.099
Large	0.2 [0.2-0.3]	0.3 [0.2-0.3]	0.158
Medium	9.1 [8.4-9.9]	8.3 [7.5-9.5]	0.079
Small	16.2 [13.9-18.7]	14.6 [12.3-16.5]	0.096
**Size (nm)**	** **	** **	** **
VLDL size	42.3 [42.0-42.4]	42.2 [42.1-42.4]	0.877
LDL size	21.0 [20.9-21.2]	20.9 [20.8-21.0]	0.112
HDL size	8.3 [8.2-8.3]	8.3 [8.3-8.3]	0.316
**Composition**			
Ratio VLDL-TG/VLDL-C	3.7 [3.5-4.1]	4.0 [3.5-4.2]	0.267
Ratio IDL-TG/IDL-C	1.2 [1.1-1.4]	1.1 [1.1-1.2]	0.064
Ratio LDL-TG/LDL-C	0.1 [0.1-0.1]	0.1 [0.1-0.2]	0.007
Ratio HDL-TG/HDL-C	0.2 [0.2-0.3]	0.4 [0.3-0.5]	<0.001
**Ratio LDL-P/HDL-P**	42.1 [33.6-47.3]	49.5 [43.3-52.8]	0.006
**Non-HDL-P (nmol/L)**	1066.3 [934.9-1194.3]	1127.8 [1027.1-1317.5]	0.087

Values expressed in median and 25-75% IQR; P value calculated by non-parametric Mann-Whitney U test; Dichotomous variables (Fisher exact test); BMI, Body Mass Index; SD, Standard deviation; SBP, Systolic blood pressure; DBP, Diastolic blood pressure; HOMA-IR, Homeostatic model assessment insulin resistance.

Correlation analysis between ALT components and variables of LV geometry and function, HOMA-IR and inflammatory markers are summarized in Table 4. The ratios LDL-P/HDL-P and HDL-TG/HDL-C were the variables better correlated to cardiac changes as well as to insulin resistance and inflammatory parameters. Moreover, considering the specific subclasses of lipoprotein particles, the results indicated that large VLDL-P, small LDL-P and total HDL-P (inversely) subclasses have the best correlation to LV remodeling and systo-diastolic dysfunction, while lipoprotein particles rich in triglycerides (VLDL-P, TG-enriched HDL and cholesterol-depleted LDL) had the highest association with HOMA-IR. Lastly, total HDL-P and small LDL-P were particles related with hs-CRP values, whereas GlycA proved to be better correlated with every component of the ALT, so this inflammatory marker was chosen for the multivariable analysis. Correlation analysis between ALT and CLP components are summarized in Supplementary Table S3. Triglycerides appeared particularly associated with VLDL-P and large HDL-P.

**Table 4:** Bivariate correlations between lipoprotein particles and left ventricle structural and functional parameters, insulin resistance index, inflammatory marker and triglycerides.

	Remodeling	Systolic GLS	Early GLSR	HOMA	hs-CRP	TG
**VLDL-P (nmol/L)**						
Total	0.37*	0.30^†^	−0.28^†^	0.52*	0.23	0.96*
Large	0.39*	0.32*	−0.31*	0.53*	0.25^†^	0.91*
Medium	0.30^†^	0.12	−0.12	0.35^†^	0.08	0.91*
Small	0.38*	0.33*	−0.30^†^	0.53*	0.25^†^	0.95*
**LDL-P (nmol/L)**						
Total	0.18	0.16	−0.11	0.28^†^	0.21	0.33*
Large	−0.05	−0.08	0.13	0.06	−0.04	0.28^†^
Medium	0.07	0.05	−0.03	0.22	0.14	0.35*
Small	0.30^†^	0.32*	−0.25^†^	0.30^†^	0.34*	0.21
**HDL-P (µmol/L)**						
Total	−0.24^†^	−0.42*	0.44*	−0.33*	−0.51*	0.14
Large	0.40*	0.11	−0.23	0.25^†^	0.17	0.49*
Medium	−0.19	−0.36*	0.39*	−0.23	−0.39*	0.06
Small	−0.20	−0.36*	0.37*	−0.30^†^	−0.47*	0.13
**Size (nm)**						
VLDL size	0.06	−0.19	0.16	−0.09	−0.21	0.29^†^
LDL size	−0.25^†^	−0.25^†^	0.23	−0.07	−0.25^†^	0.17
HDL size	0.13	0.15	−0.17	0.15	0.27^†^	−0.09
**Composition**						
Ratio VLDL-TG/VLDL-C	0.05	0.19	−0.04	0.14	0.25^†^	−0.04
Ratio IDL-TG/IDL-C	−0.16	−0.20	0.22	−0.36*	−0.32*	0.45*
Ratio LDL-TG/LDL-C	0.29^†^	0.29^†^	−0.25^†^	0.47*	0.30^†^	0.65*
Ratio HDL-TG/HDL-C	0.40*	0.30^†^	−0.33*	0.52*	0.33*	0.88*
**Ratio LDL-P/HDL-P**	0.27^†^	0.42*	−0.37*	0.45*	0.51*	0.18
**Non-HDL-P (nmol/L)**	0.23	0.20	−0.15	0.33^†^	0.24	0.42*
**Remnant cholesterol (mg/dl)**	0.35*	0.23	−0.25^†^	0.46^†^	0.20	0.97*

GLS, global longitudinal strain; GLSR, global longitudinal strain rate; HOMA-IR, homeostatic model assessment insulin resistance; hs-CRP, highly-sensitive C-reactive protein; TG, triglycerides.

Spearman coefficient. Values expressed in *r*.

* *p* < 0.01.

^†^ *p* < 0.05.

**Figure 2:**
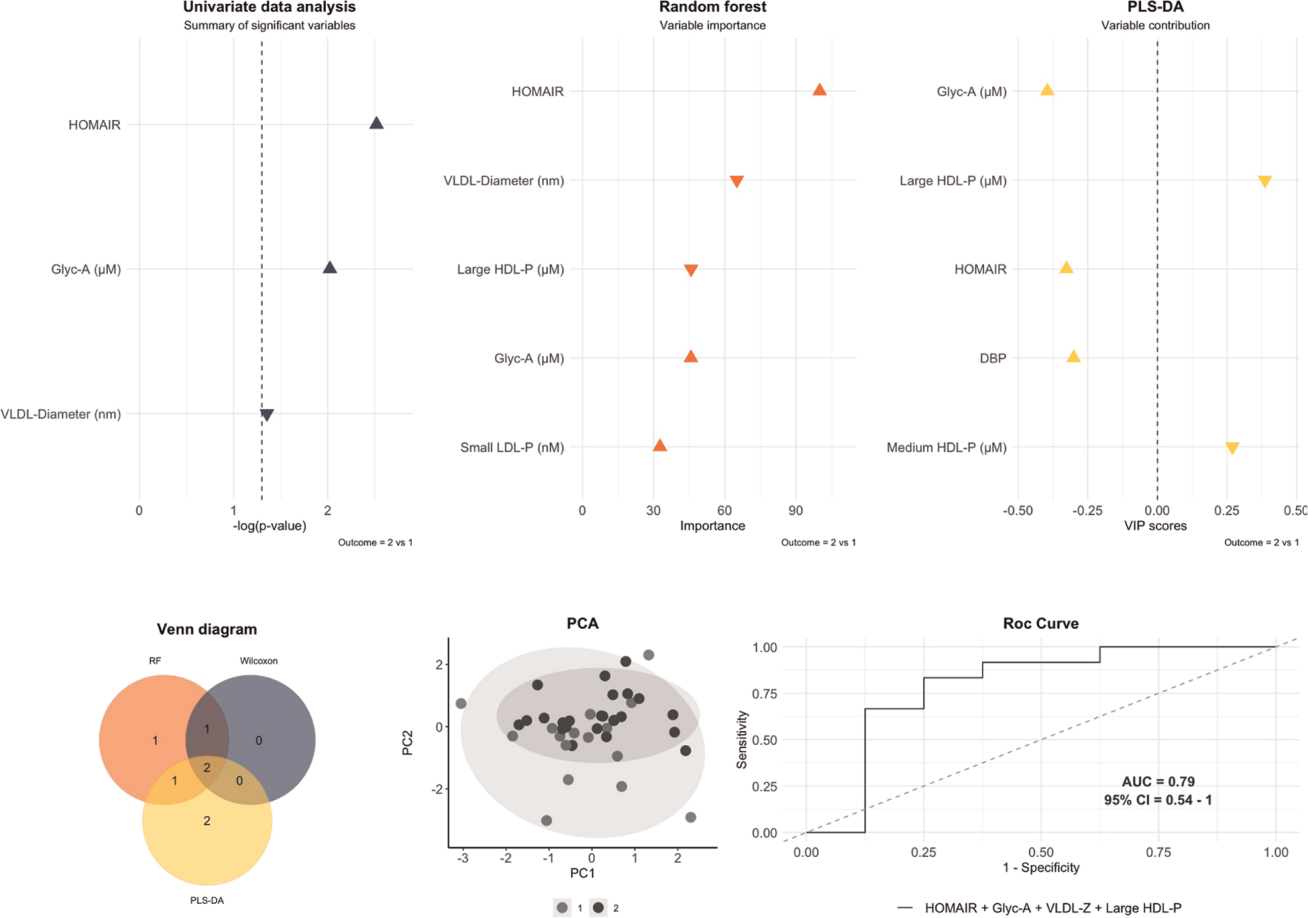
Multivariable model to differentiate mild cardiac change subjects (1) than those with severe cardiac changes (2). The variables included in the model were those that overlap by at least two of the three statistical approaches. An area under the curve (AUC) of 0.79 (95% CI: 0.54–1) was obtained with the next pattern: HOMA-IR, glycoprotein A (Glyc-A), VLDL diameter and large HDL-P. Predictive accuracy = 0.8; *p*-value = 0.051; out-of-bag error = 0.27.

**Figure 3:**
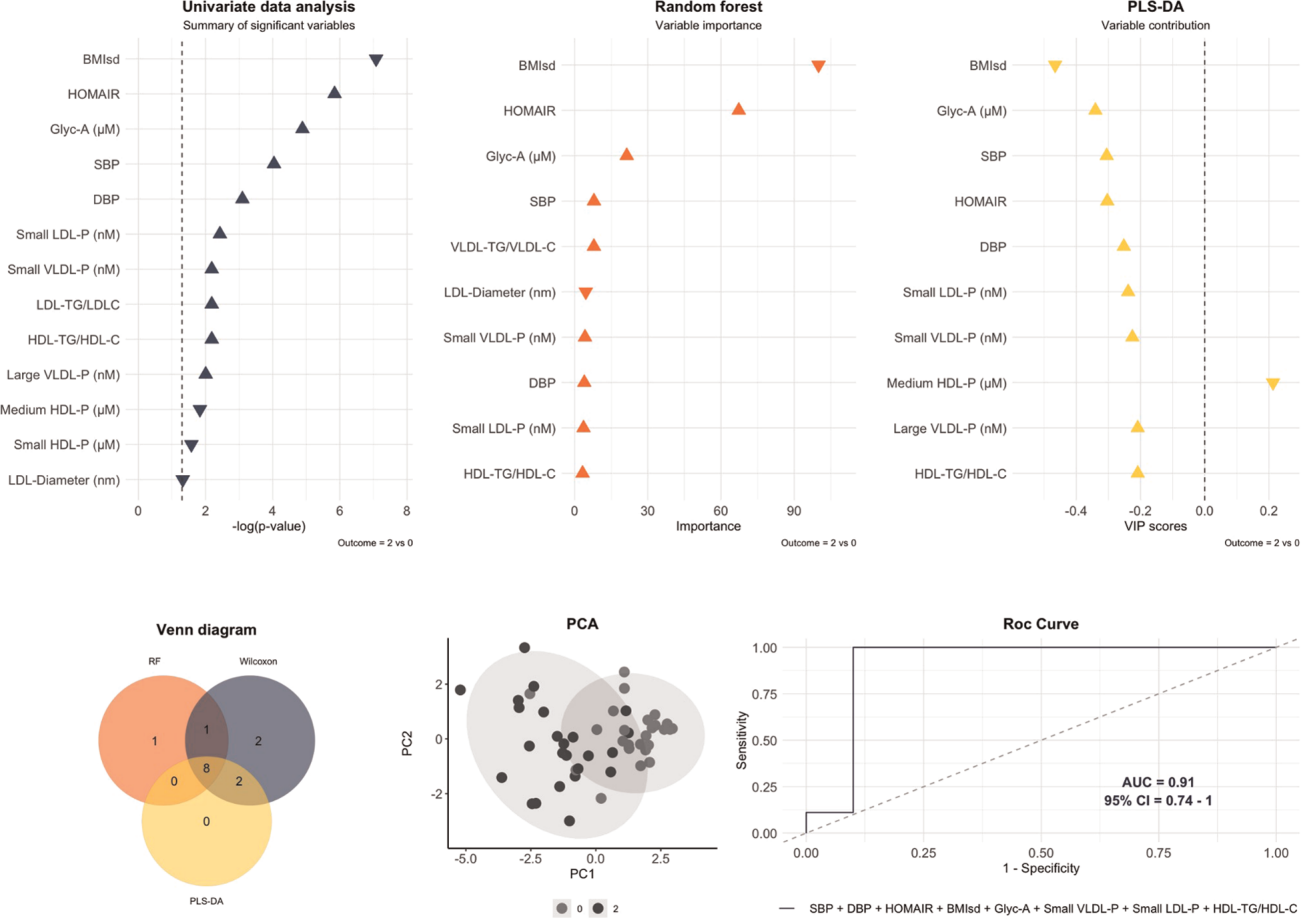
Multivariable model to differentiate normal heart subjects (0) from those with severe cardiac changes (2). The variables included in the model were those that overlap by the three statistical approaches. An area under the curve (AUC) of 0.91 (95% CI: 0.74–1) was obtained with the next pattern: body mass index standard deviation (BMIsd), HOMA-IR, systolic blood pressure (SBP), diastolic blood pressure (DBP), glycoprotein A (Glyc-A), small VLDL-P, small LDL-P and ratio HDL-TG/HDL-C. Predictive accuracy = 0.84; *p*-value = 0.004; out-of-bag error = 0.1.

**Figure 4:**
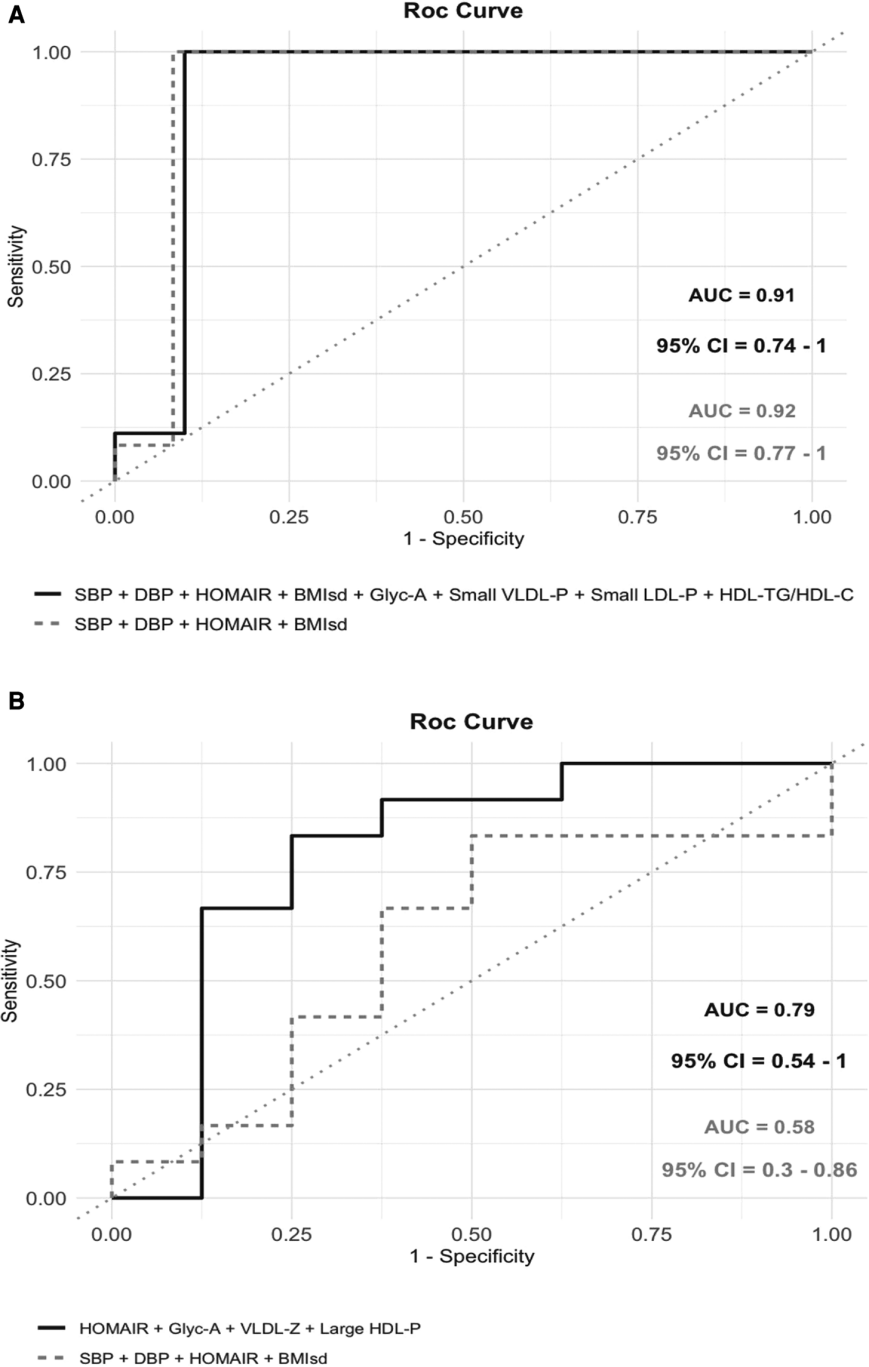
Comparative analysis between ROC curves considering only traditional cardiovascular risk factors (body mass index standard deviation, blood pressure and HOMA-IR) and ROC curves shown in Figures 2, 3. (**A**) When Normal heart subjects were compared to those with severe cardiac changes, the addition of lipoprotein subclasses and glycoprotein A to the model with traditional risk factors did not change the area under the curve (AUC) (0.92 vs. 0.91) [Only traditional risk factors: AUC 0.92 (95% CI: 0.77–1); predictive accuracy = 0.88; *p*-value = 0.0001; out-of-bag error = 0.1]. (**B**) When mild cardiac change subjects were compared to those with severe cardiac changes, the addition of lipoprotein subclasses and glycoprotein A caused an increase in the AUC (0.58 vs. 0.79) [only traditional risk factors: AUC 0.58 (95% CI: 0.3–0.86); predictive accuracy = 0.6; *p*-value = 0.596; out-of-bag error = 0.32].

The final multivariable model with the largest AUC [0.79 (95% CI: 0.54–1)] for distinguishing mild cardiac change subjects from those with severe cardiac changes identified the following pattern: HOMA-IR, GlycA, VLDL-diameter and large HDL-P (Figure 2). In contrast, for differentiating normal heart subjects from those with severe cardiac changes the largest AUC [0.91 (95% CI: 0.74–1)] resulted from the following variables: BMI standard deviation, HOMA-IR, systolic BP, diastolic BP, GlycA, small VLDL-P, small LDL-P and ratio HDL-TG/HDL-C (Figure 3). To assess the usefulness of lipoprotein subclasses and glycoprotein A in the multivariable diagnostic model, ROC curves with only traditional CV risk factors (BMI standard deviation, HOMA-IR, systolic and diastolic BP) were developed and compared with the previous analysis. Figure 4 shows the comparative ROC curve analysis. A binomial logistic regression was performed on mild vs. severe cardiac changes groups, where remaining independent parameters of the final model (adjusted *R*^2^ = 0.53, *p* < 0.0001) were HOMA-IR (OR 1.4; 95% CI: 1.0–1.9; *p* < 0.050), VLDL size (OR 0.0; 95% CI: 0.0–0.9; *p* < 0.050) and large HDL-P (OR 0.0; 95% CI: 0.0–0.3; *p* < 0.050); and on normal vs. severe cardiac changes groups, where the remaining independent parameter of the final model (adjusted *R*^2^ = 0.84, *p* < 0.0001) was the BMI standard deviation (OR 2.8; 95% CI: 1.2–6.6; *p* 0.010).

Reproducibility of echocardiographic parameters has been demonstrated previously (16), showing good intraclass correlation coefficients.

## Discussion

This study showed that morbidly obese adolescents with LV changes presented a pathological phenotype in the ALT despite exhibiting normal values in the CLP (Figure 1). Large VLDL-P, small LDL-P and total HDL-P were the subclasses more closely related to cardiac changes, while the ratio which highlighted the excess of LDL-P in relation to HDL-P appeared to be the variable more closely associated with severity in the LV changes. Furthermore, when obese adolescents were classified by MS criteria or obesity index, the resulting ALT was determined by the high triglyceridemia of these subjects, and hence, predominated TG-enriched lipoproteins, like VLDL and TG-enriched HDL (increase in the ratio HDL-TG/HDL-C) (Figure 1). Additionally, the inflammatory markers exhibited a correlation with the aforementioned pathological ALT and especially with lower levels of total HDL-P and an altered LDL subclass distribution, moved to the smallest LDL-P. Finally, multivariable models showed the relevance of VLDL size and large HDL-P—as well as BP, insulin resistance and inflammation—in the changes observed in obese adolescent's hearts. Our results have highlighted the importance of ALT and GlycA as differentiating indicators of cardiac severity in adolescents with a similar degree of obesity, where minimum differences can be found in BMI, BP or insulin resistance levels.

Previous studies performed in obese or diabetic children have shown similar results. Thus, the most frequently reported ALT was defined by increases of small LDL-P and VLDL-P and decreases of LDL size and large-medium HDL-P (13, 31–33).

VLDL are the main carriers of plasmatic triglycerides, and hence appear particularly increased in subjects with hypertriglyceridemia. In contrast, LDL are cholesterol-enriched lipoproteins and their concentration is not so triglyceride-dependent, however in hypertriglyceridemia conditions the LDL particles have been found to be smaller and compositionally cholesterol-depleted (34). The small LDL-P possesses an increased atherogenicity – on account of mechanisms like endothelial barrier crossing facility or oxidation susceptibility (34) – and it has been postulated as a predictor risk factor for coronary heart disease (35). Similarly, the relevance of LDL-P concentration to predict future CVD events rather than LDL-C has been also documented in the Framingham Offspring Study (6), where the highest risk was attributed to subjects with high LDL-P and low LDL-C. Our study, performed in a population with low LDL-C, has shown a significant increase in the small LDL-P subclass in the obese group. Additionally, in the descriptive statistics between subjects with severe cardiac changes and those with mild cardiac changes, the small LDL-P and cholesterol-depleted LDL (increase in the ratio LDL-TG/LDL-C) seemed to be the best differentiating ALT parameters, having also been found correlated with LV remodeling, systo-diastolic dysfunction, insulin resistance and inflammation. Concerning triglyceride levels, a strong positive correlation was found with all subclasses of VLDL-P and large HDL-P, but apparently, their levels were independent of the small LDL-P, as reported in the Framingham Offspring Study. Regarding VLDL-P, greater significant differences were noted in adolescents with obesity and metabolic syndrome, especially in large and small subclasses, and VLDL size seemed to be useful to differentiate levels of cardiac involvement. The hypersecretion of large VLDL-P, as a result of overnutrition and insulin resistance, has been previously proposed as a key pathological mechanism in atherogenic dyslipidemia (36, 37).

Cardioprotective functions of HDL include, in addition to the reverse cholesterol transport to the liver, the inhibition of LDL oxidation and anti-inflammatory actions in the endothelium (38, 39). However, in conditions of hypertriglyceridemia and insulin resistance, the overstimulated cholesteryl-ester-transfer protein enriches HDL composition in TG by means of exchanging TG by cholesteryl esters with other lipoprotein subclasses (40–42). Consequently, TG-enriched HDL have their beneficial features reduced – in T2-DM patients by up to 52% of the HDL antioxidative capacity (43) – and have been associated with atheroma plaque formation (44). Our results have highlighted the prevalence of TG-enriched HDL among participants with cardiac changes and especially in those with MS, as well as a relationship between TG-enriched HDL and LDL and the insulin resistance. Hence, the HDL's cardioprotective function might be decreased in these subjects.

Furthermore, a reduced number of total and medium HDL-P concentration and size has been associated with higher CVD risk in adults (45, 46), and previous studies conducted in obese adolescents with insulin resistance or T2-DM noted a significant decrease in large HDL-P (47). Nevertheless, these findings were not reproduced in the descriptive analysis of our cohort, where the significant reduction was shown in the total HDL-P concentration, without significant distinctions between the subclasses, however the large HDL-P reduction was identified as a differentiating factor between mild and severe cardiac changes in the multivariable analysis, which gives prominence to the widely recognized role of smaller HDL size in CVD (48).

Remnant cholesterol marks the overall load of TG-enriched lipoproteins (VLDL and IDL), which in situations of hypertriglyceridemia can carry as much or more cholesterol than LDL (49). How it interacts in the atherosclerosis physiopathology is still unclear but its association with CVD, as a factor related to coronary artery disease, has been established and an increased risk – up to 2.7 times in concentrations ≥39 mg/dl – has been reported as independent of obesity (5). The highest remnant cholesterol values in the present study were near to this hypothetical threshold and belonged to obese adolescents with metabolic syndrome, who also were the subjects with the highest triglyceride levels. However, a close relationship with cardiac changes was not demonstrated.

This study has some limitations. The cross-sectional design and small sample size limit the extraction of causal conclusions. The criteria for the degrees of cardiac involvement have been defined by the authors and have not been previously tested in other publications. They are based on the concept that severity increases with the addition of cardiac changes, from only LV remodeling to systolic dysfunction, defined by GLS, a widely studied parameter linked to mortality (50). The diastolic function was not included in the group definition criteria because of the lack of a single parameter for its identification, but the early GLSR was incorporated to the correlation analysis instead.

In conclusion, our results have shown that morbidly obese adolescents present an atherosclerotic ALT despite showing no pathological concentrations in the conventional lipid profile. Subjects that meet the criteria for metabolic syndrome present overall worse ALT and remnant cholesterol values because of the highest triglyceride levels.

Furthermore, when the obese adolescents are classified by the degree of cardiac change, ALT and GlycA appear to be more reliable indicators of severity than traditional CV risk factors as BMI, BP or insulin resistance. Particularly, the overage of LDL-P compared to HDL-P and the increase in small LDL-P with cholesterol-depleted LDL particles appear to be the key ALT's parameters involved in LV changes.

## Data Availability

The raw data supporting the conclusions of this article will be made available by the authors, without undue reservation.
