# Supplementary material for: Different profiles of lipoprotein particles associate various degrees of cardiac involvement in adolescents with morbid obesity
Source: Front Pediatr. 2022 Nov 22;10:887771. doi: 10.3389/fped.2022.887771 (PMC9723388; doi:10.3389/fped.2022.887771)
Supplement: Supplementary file 1 [file Datasheet1.pdf]

SUPPLEMENTAL MATERIAL

Table S1: Echocardiographic data of cardiac involvement groups

Variable	No cardiac disorder (N=25)	Mild cardiac changes (N=17)	Severe cardiac changes (N=25)	P value		
				No vs Mild	No vs Severe	Mild vs Severe
**LV mass index (g/ht$^{2.7}$)**	30.7 [26.6-35.7]	46.3 [39.4-57.4]	48.1 [44.4-54.6]	<0.001	<0.001	0.434
**RWT**	0.32 [0.30-0.36]	0.40 [0.34-0.45]	0.40 [0.35-0.43]	<0.001	<0.001	0.729
**LV remodeling (%)**	0	88	100	<0.001	<0.001	0.158
**Systolic GLS (%)**	-20.9 [-23.1 to -18.9]	-17.5 [-18.7 to -17.0]	-14.4 [-16.1 to -13.4]	0.001	<0.001	<0.001
**Early diastolic GLSR (1/s)**	3.1 [2.8-3.3]	2.3 [2.1-2.6]	2.0 [1.8-2.2]	<0.001	<0.001	0.012

Values expressed in median and 25-75% IQR; P value calculated by non-parametric Mann-Whitney U test; Dichotomous variables (Fisher exact test); LV, Left ventricle; RWT, Relative wall thickness; GLS, Global longitudinal strain; GLSR, Global longitudinal strain rate.

Table S2: Clinical and laboratory characteristics of subjects depending on metabolic syndrome (MS) diagnosis, considering only obese subjects.

Variable	No MS (<3 factors) (N=28)	MS (≥3 factors) (N=14)	P value
**Age (years)**	14 [13-15]	14 [13-15]	0.906
**BMI (kg/m²)**	36.8 [33.9-38.6]	38.1 [35.9-43.0]	0.119
**BMI SD**	7.3 [6.2-8.2]	7.9 [6.5-10.3]	0.348
**SBP (mmHg)**	120 [117-128]	131 [126-144]	0.001
**DBP (mmHg)**	70 [63-77]	77 [69-80]	0.163
**LABORATORY PARAMETERS**			
Fasting glucose (mg/dL)	83 [72-89]	84 [82-89]	0.238
HbA1c (%) (N=32)	5.3 [5.2-5.5]	5.4 [5.1-5.6]	0.833
HOMA-IR	4.3 [3.5-6.5]	7.4 [4.2-8.9]	0.043
**Classical lipid profile**			
Total Cholesterol (mg/dL)	176 [154-195]	188 [164-207]	0.218
LDL-C (mg/dL)	108 [90-118]	105 [95-121]	0.742
HDL-C (mg/dL)	44 [42-51]	42 [36-47]	0.107
Triglycerides (mg/dL)	80 [66-111]	124 [103-163]	0.002
Remnant cholesterol (mg/dL)	20.8 [15.3-27.3]	30.3 [25.2-44.4]	0.005
**Inflammatory markers**			
Highly sensitive C-reactive protein (mg/dL)	0.19 [0.11-0.24]	0.37 [0.15-0.50]	0.133
Glycoprotein A (µmol/L)	759 [700-906]	868 [777-999]	0.054

**LIPOPROTEIN PARTICLES**			
**VLDL-P (nmol/L)**			
Total	34.0 [28.5-53.8]	59.6 [47.6-82.5]	0.001
Large	0.9 [0.8-1.5]	1.6 [1.3-1.8]	0.002
Medium	3.9 [2.3-5.4]	6.1 [4.5-9.4]	0.002
Small	29.2 [25.4-45.1]	52.2 [40.1-71.4]	0.001
**LDL-P (nmol/L)**			
Total	1007.9 [960.3-1183.5]	1092.5 [998.0-1256.9]	0.518
Large	165.1 [138.4-183.2]	161.1 [147.8-180.5]	0.664
Medium	327.5 [236.8-388.5]	347.7 [256.5-377.1]	0.823
Small	608.1 [546.1-656.4]	644.2 [518.6-714.6]	0.321
**HDL-P (µmol/L)**			
Total	24.1 [21.7-26.4]	23.5 [20.3-26.0]	0.626
Large	0.2 [0.2-0.2]	0.3 [0.2-0.3]	0.626
Medium	9.0 [8.2-9.5]	8.3 [7.5-9.5]	0.308
Small	14.8 [12.9-17.0]	14.6 [12.3-16.5]	0.607
**Size (nm)**			
VLDL size	42.2 [41.9-42.3]	42.2 [42.1-42.4]	0.702
LDL size	21.0 [20.8-21.1]	20.9 [20.8-21.0]	0.296
HDL size	8.2 [8.2-8.3]	8.3 [8.3-8.3]	0.742
**Composition**			
Ratio VLDL-TG/VLDL-C	3.7 [3.5-4.0]	4.0 [3.5-4.2]	0.501
Ratio IDL-TG/IDL-C	1.2 [1.0-1.3]	1.1 [1.1-1.2]	0.308

Ratio LDL-TG/LDL-C	0.1 [0.0-0.1]	0.1 [0.1-0.2]	0.085
Ratio HDL-TG/HDL-C	0.2 [0.2-0.3]	0.4 [0.3-0.5]	0.002
**Ratio LDL-P/HDL-P**	45.0 [37.6-51.2]	49.5 [43.3-52.8]	0.147
**Non-HDL-P (nmol/L)**	1127.0 [974.2-1122.4]	1127.8 [1027.1-1317.5]	0.296

Values expressed in median and 25-75% IQR; P value calculated by non-parametric Mann-Whitney U test; Dichotomous variables (Fisher exact test); BMI, Body Mass Index; SD, Standard deviation; SBP, Systolic blood pressure; DBP, Diastolic blood pressure; HOMA-IR, Homeostatic model assessment insulin resistance.

Table S3: Bivariate Correlations between lipoprotein particles and components of conventional lipid profile.

	**Total Cholesterol**	**LDL-C**	**HDL-C**	**TG**	**Remnant cholesterol**
**VLDL-P (nmol/L)**					
Total	0.43*	0.16	-0.29†	0.96*	0.95*
Large	0.36*	0.11	-0.33*	0.91*	0.90*
Medium	0.40*	0.12	-0.18	0.91*	0.89*
Small	0.42*	0.15	-0.29†	0.95*	0.94*
**LDL-P (nmol/L)**					
Total	0.87*	0.96*	0.02	0.33*	0.36*
Large	0.83*	0.87*	0.17	0.28†	0.33*
Medium	0.83*	0.89*	0.06	0.35*	0.37*
Small	0.61*	0.71*	-0.09	0.21	0.21
**HDL-P (µmol/L)**					
Total	0.36*	0.02	0.86*	0.14	0.11
Large	0.49*	0.19	0.04	0.49*	0.48*
Medium	0.32*	-0.03	0.70*	0.06	0.03
Small	0.31*	0.05	0.75*	0.13	0.11
**Size (nm)**					
VLDL size	0.05	-0.07	0.04	0.29†	0.29†
LDL size	0.41*	0.39*	0.25†	0.17	0.21
HDL size	-0.06	-0.04	-0.31*	-0.09	-0.06
**Composition**					
Ratio VLDL-TG/VLDL-C	-0.13	0.36	-0.13	-0.04	-0.19
Ratio IDL-TG/IDL-C	-0.54*	-0.48*	0.17	0.45*	-0.51*
Ratio LDL-TG/LDL-C	0.40*	0.21	-0.18	0.65*	0.64*
Ratio HDL-TG/HDL-C	0.23	-0.03	-0.37*	0.88*	0.85*
**Ratio LDL-P/HDL-P**	0.37*	0.66*	-0.57*	0.18	0.22
**Non-HDL-P (nmol/L)**	0.89*	0.93*	-0.01	0.42*	0.44*

Spearman coefficient. Values expressed in r. *p<0.001. †p<0.050. LDL, Low-density Lipoprotein; HDL, High-density Lipoprotein; TG, triglycerides.
